# Investigation of physician burnout and the development of symptoms of anxiety and depression: burnout in consultant doctors in Ireland study (BICDIS)

**DOI:** 10.1192/bjo.2021.103

**Published:** 2021-06-18

**Authors:** Genevieve Crudden, Anne Doherty, Fabio Margiotta, Dara Byrne

**Affiliations:** 1 Central Mental Hospital, Psychiatry Department, University Hospital Galway; 2 Mater Misericordiae University Hospital, University College Dublin; 3 University Hospital Galway; 4 National University of Ireland, Galway, University Hospital Galway

## Abstract

**Aims:**

The objectives of this study were to investigate burnout in a sample of Irish Hospital Consultants and its association with psychopathology (symptoms of depression and anxiety). We examined the effect of personality factors on the development of psychopathology in response to burnout and in relation to work-related stress among the participants.

**Method:**

This is a cross-sectional survey, utilising validated psychometric measures. Self-reported online questionnaires were distributed to all hospital consultants registered with the Irish Hospital Consultants Association distribution list and were completed between September to December 2016. Questionnaires sought to determine demographic information; work-related characteristics; burnout related phenomena: emotional exhaustion, depersonalization, and a reduced sense of personal effectiveness (Maslach Burnout Inventory [MBI-GS]); symptoms of depression and anxiety (Depressive Anxiety Stress Scale [DASS]; and personality characteristics (Big Five Inventory [BFI-10]).

**Result:**

A total of 477 hospital consultants (Male = 56.6%) from hospitals in Ireland took part in the study. Of those studied, 42% reported high levels of burnout. The Depression and Anxiety Stress Scale revealed that Consultants were experiencing high levels of stress symptoms but comparatively low levels of anxiety symptoms. The study population scored highest on the conscientiousness and agreeableness subscales and lowest on the neuroticism subscale. Those who scored higher in the neuroticism subtype appeared to be at an increased risk of burnout.

**Conclusion:**

The prevalence of work-related burnout in consultants is of concern. The psychological burden of burnout is reflected in reported symptoms of stress and depression. Personality, particularly conscientiousness and agreeableness appears to impact the development of physician burnout. Strategies that modulate the relationship between personality and burnout may be beneficial for optimal health care delivery. Further research is needed to identify appropriate short and long-term strategies to ensure physician wellbeing and optimal delivery of patient care.

